# Supplementary material for: Optimized Deep Brain Stimulation Surgery to Avoid Vascular Damage: A Single-Center Retrospective Analysis of Path Planning for Various Deep Targets by MRI Image Fusion
Source: Brain Sci. 2022 Jul 22;12(8):967. doi: 10.3390/brainsci12080967 (PMC9332267; doi:10.3390/brainsci12080967)
Supplement: Supplementary file 1 [file brainsci-12-00967-s001.zip › Supplementary Files/Figure S1. Comparisons of Ring and Arc Angle Values of the Trajectories Between the no ICH Cases and ICH Cases.pdf]

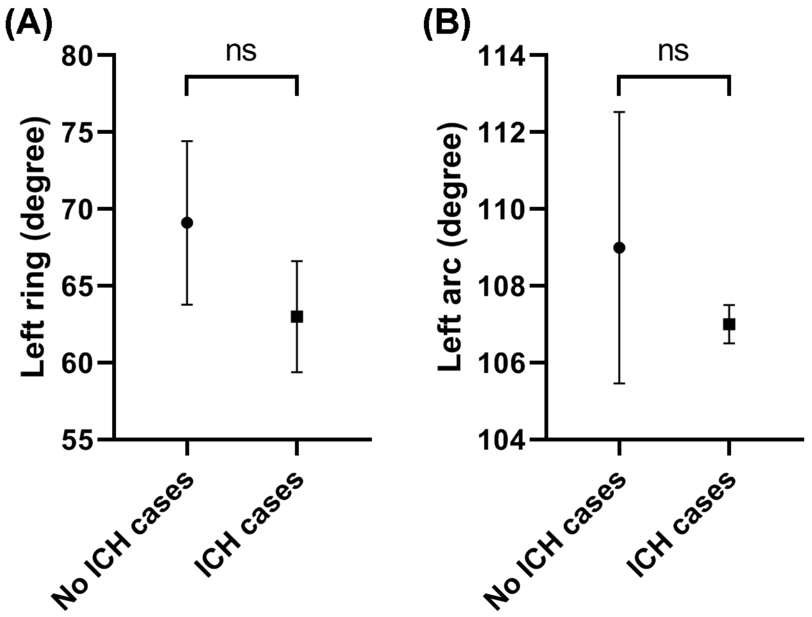


**Figure S1.** The comparisons of ring and arc angle values of the trajectories for STN lead placement between the no ICH cases and ICH cases (mean±SD). (**A**) Left ring: $p$ = 0.0518 (No ICH cases: 69.11±5.33; ICH cases: 63±3.61). (**B**) Left arc: $p$ = 0.3324 (No ICH cases: 109.00±3.53; ICH cases: 107.00±0.50). ns, no significant difference.
